# Putative sugarcane *FT/TFL1* genes delay flowering time and alter reproductive architecture in Arabidopsis

**DOI:** 10.3389/fpls.2014.00221

**Published:** 2014-05-26

**Authors:** Carla P. Coelho, Mark A. A. Minow, Antonio Chalfun-Júnior, Joseph Colasanti

**Affiliations:** ^1^Setor de Fisiologia Vegetal, Departamento de Biologia, Universidade Federal de LavrasLavras, Brazil; ^2^Department of Molecular and Cellular Biology, University of GuelphGuelph, ON, Canada

**Keywords:** *Saccharum* spp., bioenergy crop, floral induction, *FT*-like genes, florigen orthologs, PEBP family

## Abstract

Agriculturally important grasses such as rice, maize, and sugarcane are evolutionarily distant from Arabidopsis, yet some components of the floral induction process are highly conserved. Flowering in sugarcane is an important factor that negatively affects cane yield and reduces sugar/ethanol production from this important perennial bioenergy crop. Comparative studies have facilitated the identification and characterization of putative orthologs of key flowering time genes in sugarcane, a complex polyploid plant whose genome has yet to be sequenced completely. Using this approach we identified phosphatidylethanolamine-binding protein (PEBP) gene family members in sugarcane that are similar to the archetypical *FT* and *TFL1* genes of Arabidopsis that play an essential role in controlling the transition from vegetative to reproductive growth. Expression analysis of *ScTFL1*, which falls into the *TFL1*-clade of floral repressors, showed transcripts in developing leaves surrounding the shoot apex but not at the apex itself. *ScFT1* was detected in immature leaves and apical regions of vegetatively growing plants and, after the floral transition, expression also occurred in mature leaves. Ectopic over-expression of *ScTFL1* in Arabidopsis caused delayed flowering in Arabidopsis, as might be expected for a gene related to *TFL1*. In addition, lines with the latest flowering phenotype exhibited aerial rosette formation. Unexpectedly, over-expression of *ScFT1*, which has greatest similarity to the florigen-encoding *FT*, also caused a delay in flowering. This preliminary analysis of divergent sugarcane *FT* and *TFL1* gene family members from *Saccharum spp.* suggests that their expression patterns and roles in the floral transition has diverged from the predicted role of similar PEBP family members.

## Introduction

Flowering time is a crucial and highly controlled mechanism in plants that has a direct impact on reproductive success and survival (Imaizumi and Kay, 2006). Moreover, the floral transition in crop plants is directly related to crop yield. In order to survive imminent seasonal changes, plants have developed core signaling pathways that integrate day-length perception with developmental reprogramming. Signals are initiated outside of the shoot apical meristem (SAM) and a response cascade is triggered, ultimately reaching the SAM where cellular changes occur, leading to the formation of reproductive structures instead of leaves. The study of flowering time mutants in Arabidopsis has been instrumental in defining six signaling pathways: photoperiodic, autonomous, vernalization, gibberellin, ambient temperature and age-dependent control (Fornara et al., 2010). FLOWERING LOCUS T (FT)/TERMINAL FLOWER 1 (TFL1) are phosphatidylethanolamine-binding protein (PEBP) family members that are similar to mammalian PEBPs (Banfield et al., 1998; Ahn et al., 2006). In Arabidopsis, TFL1 is responsible for maintaining the inflorescence in an indeterminate state, with loss of TFL1 function resulting in the production of terminal flowers (Bradley et al., 1997). Although the *TFL1* gene sequence is highly similar to *FT*, *TFL1* acts antagonistically by delaying floral commitment (Hanzawa et al., 2005; Ahn et al., 2006). Whereas the FT protein interacts with the FLOWERING LOCUS D (FD) bZIP transcription factor at the SAM to promote flowering (Abe et al., 2005; Wigge et al., 2005), TFL1 protein similarly binds to FD to repress downstream genes such as *APETALA 1 (AP1)* and LEAFY (*LFY)* in the central zone of the meristem (Ratcliffe et al., 1999; Hanano and Goto, 2011). Opposite functions of *TFL1* and floral meristem genes reflect their specific expression in separate domains. *TFL1* is expressed in central cells of the SAM whereas the floral meristem genes are concentrated in the peripheral cells (Mandel et al., 1992; Kempin et al., 1995; Bradley et al., 1997). When floral meristem identity gene expression is reduced, flowers have shoot-like characteristics (Irish and Sussex, 1990; Schultz and Haughn, 1991, 1993; Huala and Sussex, 1992; Weigel et al., 1992; Bowman et al., 1993; Shannon and Meekswagner, 1993). Upon floral transition, *TFL1* is up-regulated to maintain indeterminate inflorescence meristem and to counterbalance *FT* activity (Shannon and Meekswagner, 1991; Bradley et al., 1997; Ratcliffe et al., 1999; Conti and Bradley, 2007; Hanano and Goto, 2011; Jaeger et al., 2013).

Several structural and biochemical features of FT protein support the hypothesis that FT is a major component of the florigen that triggers floral evocation at the SAM (Taoka et al., 2013). *FT* is expressed in phloem-specific tissues under floral inductive long-day conditions (Takada and Goto, 2003; An et al., 2004) and is able to traffic long distances intercellularly from companion cells to the SAM (Jaeger and Wigge, 2007; Mathieu et al., 2007). Characterization of FT homologs that induce flowering in diverse species suggests that FT is a highly conserved florigen (Kojima et al., 2002; Lifschitz et al., 2006; Corbesier et al., 2007; Lin et al., 2007; Tamaki et al., 2007; Lazakis et al., 2011; Meng et al., 2011). For example, the rice FT ortholog, Heading date3 (Hd3a), is a mobile signal synthesized in leaves that is capable of reaching the SAM (Kojima et al., 2002; Tamaki et al., 2007); Zea mays *CENTRORADIALIS8* (*ZCN8*) gene is expressed in the leaf and is able to induce flowering in Arabidopsis *ft* mutants when expressed under the control of a phloem-specific promoter (Lazakis et al., 2011; Meng et al., 2011); the tomato *SINGLE FLOWER TRUSS* (*SFT*) dependent graft-transmissible elements complement developmental defects in *sft* mutants and substitute long-day conditions in Arabidopsis (Lifschitz et al., 2006). In addition, the *Beta vulgaris* floral inducer *FT2 (BvFT2)* is needed for normal flower initiation in sugar beet (Pin et al., 2010).

Plants typically have more than one *FT* related homolog, and domain analysis suggests that variation in specific regions of the FT protein are responsible for alternative functions, such as floral repression (Hanzawa et al., 2005; Ahn et al., 2006; Pin et al., 2010; Blackman et al., 2011; Harig et al., 2012). These observations, and comparison of FT function in various plant species suggests that the ancestor of *FT* is a floral repressor (Karlgren et al., 2011; Harig et al., 2012). Augmenting the acknowledged role of FT in flowering time, recent discoveries associate FT function with other meristem-related mechanisms (Bohlenius et al., 2006; Shalit et al., 2009; Navarro et al., 2011), consolidating FT as a key mobile signal that is not only related to floral transition but also related to diverse developmental events in plants.

Perennial plants depend on the maintenance of growth through several seasons, balancing nutritional status, biomass accumulation, and alternating vegetative and reproductive growth over the years. Flowering time genes are largely conserved between annual and perennial plants (Albani and Coupland, 2010), however perennial plants also must account for plant age to coordinate competence to flower. In the perennial *Arabis alpina*, sensitivity to vernalization depends on plant age; a condition which the PEBP member gene, *AaTFL1*, sets as a threshold to control the age-dependent pathway to flowering (Wang et al., 2011; Bergonzi et al., 2013). In perennial sugarcane, a qualitative short-day plant, little is known about the genetic control of floral induction. The transition to reproductive growth is undesirable in commercial sugarcane cultivars because the production of floral structures redirects carbon assimilates from stalks to inflorescences and results in loss of accumulated sucrose (Berding and Hurney, 2005). Therefore understanding the genetic underpinnings of flowering time in sugarcane provides a basis for the development of new strategies to improve agronomic traits such as increased biomass and sugar production in this important food and bioenergy crop. Here we isolate and characterize two novel sugarcane PEBP members and show that they alter flowering time and floral architecture in Arabidopsis.

## Materials and methods

### Plant growth conditions and genotyping

Sugarcane plants, variety RB72 454, were grown in a greenhouse under either 14-h long-day conditions at 27°C with 10-h nights at 22°C, or 12-h short-day inductive conditions representing field conditions with 20-20-20 NPK fertilizer supplemented with micronutrients added as required. Arabidopsis plants, ecotype *Columbia (Col-0)*, were cultivated in Conviron growth chambers under conditions of 16-h days at 23°C with 8-h nights at 21°C, with a light intensity of 120 μmolm^−2^s^−1^ and 60% humidity. Arabidopsis plants with segregating transgenes were genotyped using the Sigma REDExtract-N-Amp Plant PCR Kit (Sigma Biosciences) following manufacturer's instructions, and PCR was performed using kanamycin primers - KanrF: 5'-ATACTTTCTCGGCAGGAGCA-3' and KanrR: 5'-ACAAGCCGTTTTACGTTTGG-3'.

### Isolation and cloning of *FT/TFL1* homologs from sugarcane leaves

Mature and immature leaf tissues from sugarcane plants under inductive and non-inductive conditions were collected for total RNA extraction (TRIzol Reagent) and genomic DNA as previously described (Colasanti et al., 1998). For RNA assays, complementary cDNA was synthesized using the qScript cDNA SuperMix (Quanta Bioscences) according to the manufacturer's instructions. Sequences were amplified using specific primers designed at the UTR region of the genes: *ScTFL1F*: 5'-GTCCGATTAGCTTGCTGCAT-3'; *ScTFL1R*: 5'-GGCCATGCTCATAACTTTGG-3'; *ScFT1F*: 5'-ATATGGCTAATGACTCCCTGACG-3'; *ScFT1R*: 5'-CTGGACATGAGGGGTAGGTAAAT-3'. Genomic and complementary *ScTFL1/ScFT1* sequences were cloned to the CloneJET PCR Cloning Gene (Thermo Scientific) and sequenced.

### Phylogenetic analysis of the ScTFL1 and ScFT1 candidates with orthologs of related species

Deduced amino acid sequences of sugarcane ScTFL1/ScFT1 compared to homologs from other species were aligned with translated sequences for Arabidopsis *TFL1* and *FT; ZCN1, ZCN2*, and *ZCN8* (maize); *RCN1* and *Hd3a* (rice); *NtFT1* to *NtFT4* (tobacco); and *BvFT1* and *BvFT2* (sugar beet), using the software BioEdit 7.1.3.0 (Hall, 1999). Phylogenetic trees were constructed by MEGA software, version 4.0 (Tamura et al., 2007), with the neighbor-joining comparison model (Saitou and Nei, 1987), *p*-distance method and pair-wise deletion. Bootstrap values from 1000 replicates were used to assess the robustness of the trees (Felsenstein, 1985). Phylogenetic analysis that included the deduced amino acid sequences of incomplete sugarcane genes was corrected by deleting positions with gaps from the alignment. Gene structure information for homologs was accessed at the Phytozome 9.1 genome database available online (www.phytozome.net).

### Construction of overexpression vector and arabidopsis transformation

Candidate genes for *TFL1* and *FT* amplified from sugarcane leaf RNA were cloned into Gateway entry vector pDONR-221 using the BP recombination reaction and the subsequent products were recombined with the destination vector pK2GW7 by a LR clonase originating from the expression vector 35S::*ScTFL1* and 35S::*ScFT1*. Gateway sites were added to the sequencing primers for cloning purposes as follows:

ScTFL1gatF: 5'-GGGGACAAGTTTGTACAAAAAAGCAGGCTGTCCGATTAGCTTG-CTGCAT-3' and ScTFL1gatR: 5'-GGGGACCACTTTGTACAAGAAAGCTGGGTGGCCATG-CTCATAACTTTGG-3' and ScFT1gatF: 5'-GGGGACAAGTTTGTACAAAAAAGCAGGC-TATATGGCTAATGACTCCCTGACG-3' and ScFT1gatR: 5'-GGGGACCACTTTGTACAA-GAAAGCTGGGTCTGGACATGAGGGGTAGGTAAAT-3'. *Agrobacterium tumefaciens* strain GV3101::pMP90 containing the over-expression constructs were introduced into Arabidopsis plants by floral dip (Clough and Bent, 1998). *Agrobacterium* containing *ScTFL1* and *ScFT1* over-expression constructs were introduced to the Columbia (*Col-0)* ecotype. Fifty T1 individuals overexpressing *ScTFL1* and 18 T1 lines carrying the 35S::ScFT1 construct germinated on MS plates supplemented with 50 μg/ml kanamycin and resistant seedlings were transplanted to soil to obtain T2 seeds. Segregation analysis showed that 20 and 14 T2 lines, respectively, had single insertions of the 35S::ScTFL1 and 35S::ScFT1 transgenes (X2 test, *p* < 0.05). All individuals with the transgene showed the late flowering phenotype whereas segregants without the transgene flowered normally. Ten individuals from four independent single insertion lines each that were homozygous for the transgene were selected for phenotypic analyses of *ScTFL1* and *ScFT1* overexpression. Flowering time was scored by counting the number of rosette leaves at the appearance of the first floral bud in primary inflorescences.

### Gene expression analysis using semi-quantitative and quantitative RT-PCR

Gene and transgene expression analysis was carried out using semi-quantitative RT-PCR and real time RT-PCR. For *ScTFL1* and *ScFT1* expression analysis, RNA was extracted as described above from sugarcane mature leaves, immature leaves and the SAM enriched region. Complementary DNA (cDNA) was prepared using qScript cDNA SuperMix (Quanta Biosciences) according to the manufacturer's instructions. *ScTFL1* primers were designed to assess transgene expression in the *ScTFL1* transgenic lines: ScTFL1qRTF: 5'- GACTTGCGGTCTTTCTTCACA -3'; ScTFL1qRTR: 5'- AGGCATCTGTTGTCCCAGGT -3'. Expression of the *ScFT1* gene in transgenic plants was assessed by qRT-PCR analysis with the primers ScFT1qPCR-F (GGCTAATGACTCCCTGACGA) and ScFT1qPCR-R (CCATCCCTT CAAACACTGGT). PerfeCTa SYBR Green SuperMix (Quanta Biosciences) and an Applied Biosystems 7300 Real Time PCR instrument were used, and data was analyzed by the Pfaffl method with efficiency correction to obtain fold difference in expression (Pfaffl, 2001). Three biological replicates of three technical replicates were analyzed. *Actin8* (ActinrtF: 5'-GCCGATGCTGATGACATTCA-3' and ActinrtR: 5'-CTCCAGCGAATCCAGCCTTA-3') and *ScGAPDH* (ScGAPDHF: 5'-CACGGCCACTGGAAGCA-3' and ScGAPDHR: 5'-TCCTCAGGGTTCCTGATGCC-3') were used for normalization and the calibrator was the average ΔCt for the independent line with lower expression level. Statistical significance is reported by the Student's *t-test* with *P* < 0.05.

### Scanning electron microscopy (SEM)

At least three inflorescences per plant were harvested from *ScTFL1* over-expressed plants and images were captured with a Hitachi Tabletop TM-1000 Scanning Electron Microscope. Dimension bars were added using the ImageJ software (Abràmoff et al., 2004).

## Results

### Isolation of a *TFL1* homolog from *saccharum* spp. and expression analysis in different tissues

**Figure 1:**
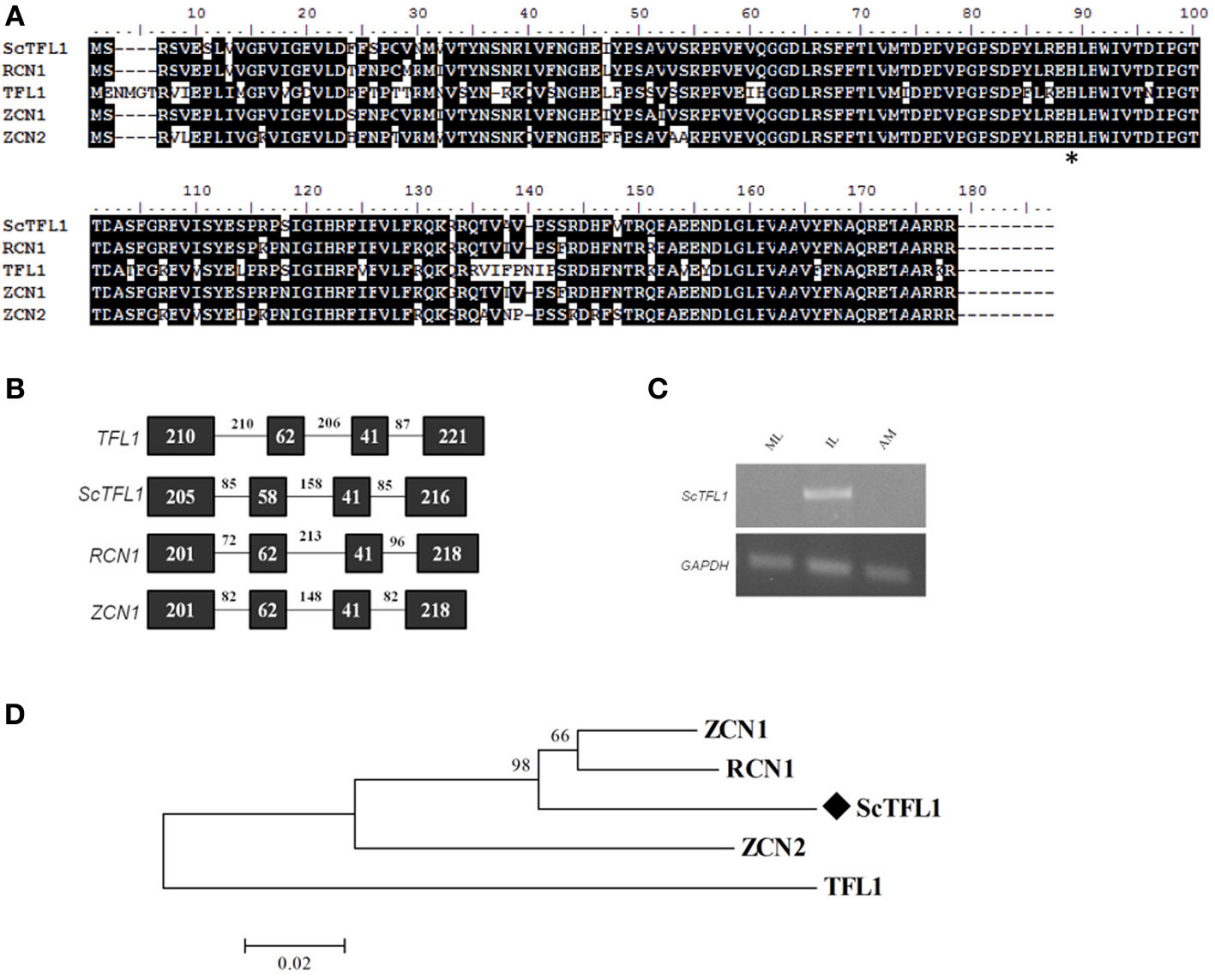
**Sequence conservation among TFL-like genes. (A)** Alignment of the ScTFL1 candidate with homologs from different species: Arabidopsis TFL1; rice RCN1; and maize ZCN1. Asterisk (^*^) highlights the amino acid residue conserved in all TFL1 homologs. **(B)** TFL1 gene structure conservation among TFL1 homologs, consisting of four exons and three introns. Boxes represent exons and lines, introns. Numbers indicate size of each exon and intron. **(C)** Expression pattern of *ScTFL1* in different tissues by semi-quantitative PCR; sugarcane *GAPDH* endogenous control was used as control. IL: apex-surrounding immature leaves; ML, mature leaves; AM, apical meristem. **(D)** Evolutionary relationship of TFL1 homologs. Amino acid sequences from different species were aligned using ClustalW, the evolutionary history was inferred using the Neighbor-Joining method (Saitou and Nei, 1987). Bootstrap values from 1000 replications were used to assess the robustness of the trees. Sugarcane TFL1 candidate gene is highlighted by a diamond symbol (♦) and TFL1 homologs from related species are deposited at the Genbank database. Accession numbers: ZCN1 (ABX11003.1), ZCN2 (ABX11004.1), RCN1 (ABA95827.1), and TFL1 (AED90661.1), ScTFL1 (KJ496328).

Candidates for *FT/TFL1* gene family members were identified in the sugarcane EST database, SUCEST (Vettore et al., 2001; Coelho et al., 2013). A complete sequence for a *TFL1-like* subfamily candidate was identified and termed *ScTFL1.* The deduced ScTFL1 protein had highest similarity to maize genes ZCN1 and ZCN2 proteins (93 and 84% amino acid identity, respectively), 92% identity to rice RCN1, and 70% identity to Arabidopsis TFL1 (Figure 1A). ScTFL1 sequence is more similar to the rice and maize homologs compared to Arabidopsis (Figure 1D). The founding member of this family, Arabidopsis *TFL1*, is highly conserved between species, and homologs have been reported in several different species (Hecht et al., 2005; Danilevskaya et al., 2010; Taylor et al., 2010; Mauro-Herrera et al., 2013).

Plant PEBP proteins are general regulators of signaling complexes, as shown by the tomato SELF PRUNNING (SFP) protein, a TFL1 homolog that acts through interaction with different proteins (Pnueli et al., 2001). The PEBP family consists of three gene subfamilies named *MOTHER-of-FT* (*MFT*)*-like*, *TFL1-like* and *FT-like* (Chardon and Damerval, 2005). In sugarcane, eight candidate PEBP gene family members were identified by *in silico* analysis in the sugarcane database and found to belong to several different subfamilies; one *MFT-like* gene, one TFL1-like and six FT-like candidates (Coelho et al., 2013). Six members of the *TFL1*-like subfamily were reported in maize, *ZCN1* to *ZCN6* (Danilevskaya et al., 2010), and four members were identified in rice: Oscen1 to Oscen4 (Nakagawa et al., 2002). Completion of the sugarcane genome sequence likely will reveal more PEBP members in this species.

Comparing the deduced ScTFL1 amino acid sequence to known TFL1-like homologs from other plant species shows that they all share a histidine residue at position 89 (H89) (Figure 1A); in Arabidopsis this residue is at a key position that determines whether TFL1 or FT act as a floral repressor or promoter, respectively (Hanzawa et al., 2005). The gene structure of *ScTFL1* is similar to the related *TFL1* orthologs, consisting of three introns and four exons of similar sizes. (Figure 1B). The fourth exon contains a specific region, segment B, that is critical for FT to function as a floral promoter or TFL1 as a floral repressor (Ahn et al., 2006). Residues Gln140, Asp144 and Glu141 (found in FT, TFL1, and BFT, respectively) of segment B may have an important function in determining FT-like or TFL1-like activity (Ahn et al., 2006; Yoo et al., 2010). This segment forms an external loop that varies in TFL1 but not in FT, and it seems that the opposite activity in flower induction is derived from hydrogen bond formation near the binding pocket in TFL1 but not in FT, suggesting that this segment is crucial for the co-activation of specific, yet-to-be identified FT/TFL1 interactors (Ahn et al., 2006; Pin et al., 2010; Taoka et al., 2011; Harig et al., 2012; Taoka et al., 2013). Consistent with this, FT has a tyrosine residue at position 85 (Y85), a key difference that specifies FT function as a floral promoter in Arabidopsis (Hanzawa et al., 2005), although in some species it has been reported that the FT-likes containing the Y85 residue may act as a floral repressor if there is variation in segment B.

We determined the expression pattern of *ScTFL1* at the vegetative apical meristem region, mature leaves and the immature leaves surrounding the meristem in 7-month old sugarcane plants. *ScTFL1* is expressed in the young leaves that enfold the meristem, however no transcript was detected in the shoot apical region. (Figure 1C). In Arabidopsis, *TFL1* is expressed in young axillary meristems and is later confined to the central core of the meristem (Conti and Bradley, 2007). The *ScTFL1* expression pattern suggests that this gene acts in regions adjacent to the meristem in vegetative sugarcane plants. Similarly, in maize, which is an annual plant, *ZCN1* and *ZCN2* are expressed in both vegetative and reproductive phases, with *ZCN1* mRNA detected in vascular bundles of leaf primordia and *ZCN2* in leaf axils of shoot apices (Danilevskaya et al., 2010).

### Ectopic expression of *ScTFL1* alters flowering time and maintains indeterminate fate of inflorescence meristems in transgenic arabidopsis plants

To understand the role played by this sugarcane *TFL1* homolog, we examined transgenic Arabidopsis plants over-expressing the *ScTFL1* driven by the constitutive 35S CaMV promoter. More than 40 transgenic lines were isolated and found to flower later than wild-type; four independent T2 lines homozygous for the transgene (ScTFL1-5; ScTFL1-6; ScTFL1-11, and ScTFL1-41) were selected for further analysis. The prolonged vegetative phase was manifested as an increase in the number of rosette leaves in all transgenic lines, ranging from 15.4 to 17.7 leaves on average, compared to the 11.4 leaves in Col-0 plants (Table 1). All four lines had ectopic expression of the ScTFL1 transgene, however the differences in flowering time did not correlate with the level of exogenous transcript (Supplemental Figure 1A). Otherwise there were no morphologic differences in vegetative structures, such as the serrated leaves that were reported from over-expression of the *BROTHER of FT and TFL1* (*BFT*) gene in Arabidopsis (Yoo et al., 2010).

**Table 1:** **Flowering characteristics of four 35S::*ScTFL1* independent transgenic lines**.

**Plant genotype**	**Days to flowering**	**Number of leaves**	**Number of plants**
*Col-0* wild-type	32	11.4 ± 0.54	5
ScTFL1-5	41	15.4 ± 1.35^a^	10
ScTFL1-6	41	14.3 ± 1.34^a^	10
ScTFL1-11	41	15.2 ± 1.73^a^	10
ScTFL1-41	47	17.7 ± 1.60^a^	10

a *Indicates statistically different from wild-type with p > 0.05 by student t-test*.

**Figure 2:**
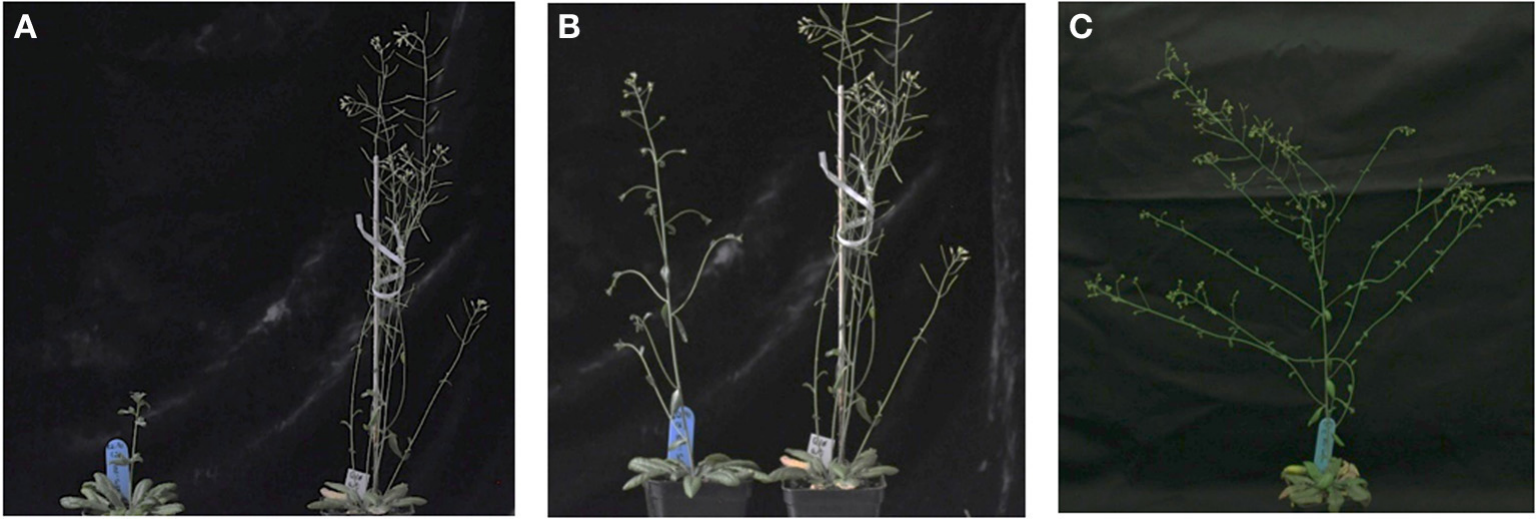
**Ectopic *ScTFL1* expression affects inflorescence architecture in transgenic Arabidopsis. (A)** Growth of 35S::ScTLF1-41 transgenic plants (left) and Col-0 (right) under long-day conditions after 50 days; and **(B)** 55 days; and **(C)** 64 days of germination, at this point Col-0 wild-type plants have completed the life cycle. All plants are in the Col-0 background.

**Figure 3:**
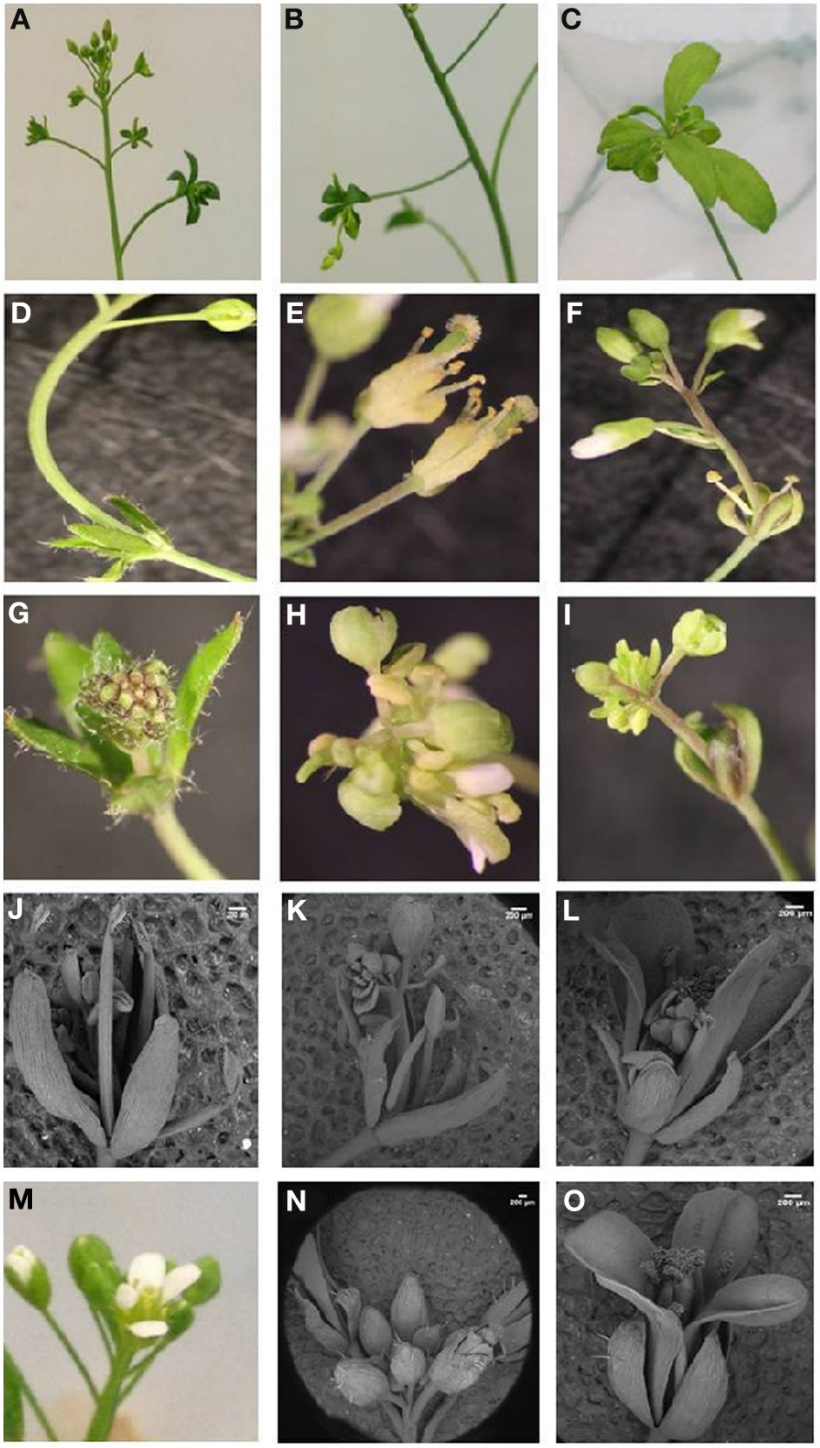
***ScTFL1* transgenic plants phenotypes. (A–D)** Examples of aerial rosettes phenotype of 35S::ScTFL1 lines. **(E–I)** Abnormal flower formation in emerging floral buds. **(J–L)** Scanning electronic microscopy (SEM) showing floral buds emerging from 35S::*ScTF1* inflorescences. **(M–O)** Wild-type flower and inflorescences. Scale bars: 200 μm.

With regard to reproductive development, ectopic expression of *ScTFL1* altered flowering time (Table 1; Supplemental Figure 2) but also affected the formation of the inflorescence structures, as typified in the most severe line, ScTFL1-41 (Figure 2A). The other late flowering lines examined, ScTFL1-5, -6 and -11, showed similar defects in reproductive architecture, although to a lesser extent than the most severe line ScTFL1-41 which was also exhibited the latest flowering. In addition ScTFL1-41 plants had a highly branched phenotype (Figures 2B,C), shoot-like inflorescences, aerial rosettes, abnormal flower formation (Figure 3), and prolonged life cycle (>64 days). A similar phenotype was reported in Arabidopsis over-expressing *TFL1* and *TFL1-like BFT*, and their respective rice and maize homologs; i.e., in developmental phases were delayed and similar effects on plant architecture were observed (Ratcliffe et al., 1998, 1999; Jensen et al., 2001; Nakagawa et al., 2002; Danilevskaya et al., 2010; Yoo et al., 2010).

### *ScFT1* is a putative FT ortholog that delays flowering in arabidopsis

**Figure 4:**
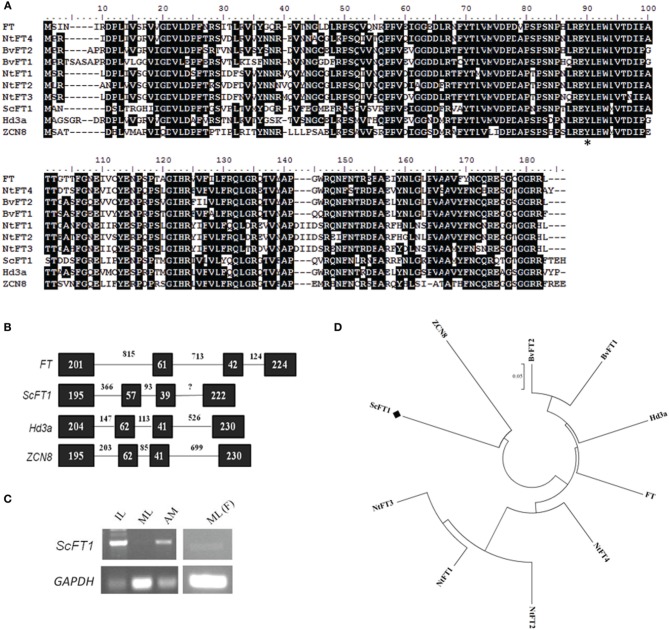
**Sequence conservation among FT-like genes. (A)** Amino acid sequence alignment of ScFT1 candidate with homologs from different species: Arabidopsis FT, rice Hd3a; and maize ZCN8; tobacco NtFT1-4; sugar beet BvFT1/2. Asterisk (^*^) highlights the amino acid residue conservation in all FT homologs. **(B)** Evolutionary relationship of TFL1 homologs. **(C)** Expression pattern of *ScFT1* of different tissues by semi-quantitative PCR, IL: apex-surrounding immature leaves; ML, mature leaves; AM, apical meristem; ML(F), mature leaves of mature flowering plants. **(D)** Amino acid sequences from different species were aligned using ClustalW, the evolutionary history was inferred using the Neighbor-Joining method (Saitou and Nei, 1987). The tree is drawn to scale, with branch lengths in the same units as those of the evolutionary distances used to infer the phylogenetic tree. Bootstrap values from 1000 replications were used to assess the robustness of the trees. Accession numbers: BvFT1 (ADM92608.1), BvFT2 (ADM92610.1), NtFT1 (AFS17369.1), NtFT2 (AFS17370.1), NtFT3 (AFS17371.1), NtFT4 (AFS17372.1), FT (BAA77838.1), Hd3a (BAB61030.1), ZCN8 (ABX11010.1), ScFT1 (KJ496327).

In parallel with the characterization of *ScTFL1*, we isolated *ScFT1* from mature sugarcane plants and compared the sequence and expression pattern to Arabidopsis *FT* and other homologs. Comparison of the ScFT1 deduced protein with FT homologs from different species (Figure 4A) showed it to be 59% identical to Arabidopsis FT, 59% to rice Hd3a; 57% to maize ZCN8; 62% to sugar beet BvFT2 and 61% to tobacco NtFT4. Sugar beet and tobacco candidates that act antagonistically to flowering had less similarity to ScFT1: NtFT1, -2, -3 were 57, 54, and 54%, respectively, and the sugar beet BvFT1, 59%.

Phylogenetic analysis of FT-like proteins showed that the FT-like floral repressors from tobacco clade together and the floral promoter NtFT4 clades with the FT-like floral promoters, FT and Hd3a (Figure 4D). As sugarcane and maize are more closely related to each other than to other species examined it is not unexpected that ScFT1 clades with maize ZCN8, but not to other FT-like proteins, such as FT and Hd3a. Despite the finding that ZCN8 does not clade with FT-like floral promoters, the strong association of *ZCN8* with a maize flowering time QTL and its ability to complement an Arabidopsis *ft* mutant suggests that it acts as a floral promoter in maize (Lazakis et al., 2011; Meng et al., 2011). Similar to all PEBP family members, the *ScFT1* gene consists of four exons and three introns, with similar exon sizes but largely varying the number of nucleotides in the introns (Figure 4B).

Expression analysis in sugarcane showed that *ScFT1* transcript is present in mature leaves of vegetative phase plants, although it was expressed in immature leaves and SAM of the same plants. Interestingly, *ScFT1* transcript was detected in mature leaves of flowering plants (Figure 4C), suggesting a possible role in post-floral transition plants.

### Transgenic plants overexpressing *ScFT1* delayed flowering and caused abnormal silique development

Sugarcane *ScFT1* was over-expressed in Arabidopsis to test whether this *FT-like* candidate is involved in controlling flowering time. Four independent transgenic lines were selected for flowering time analysis and shown to over-express the transgene (Supplemental Figure 1B). Unexpectedly, in all cases *ScFT1* over-expression resulted in late flowering plants, with an average range of 16.1–24.5 rosette leaves, compared to the 11.4 rosette leaves of Col-0 wild-type (Table 2). The most severe effect on flowering time was observed in ScFT1-3, which had a significantly higher transgene expression than the other three lines (Supplemental Figure 1B).

**Table 2:** **Flowering characteristics of four 35S::*ScFT1* independent transgenic lines**.

**Plant genotype**	**Days to flowering**	**Number of leaves**	**Number of plants**
*Col-0* wild-type	32	11.4 ± 0.54	5
ScFT1-1	37	16.1 ± 0.87^a^	10
ScFT1-2	37	17.3 ± 2.16^a^	10
ScFT1-3	46	24.5 ± 1.84^a^	9
ScFT1-4	37	19.6 ± 1.41^a^	10

a *Indicates statistically different from wild-type with p > 0.05 by student t-test*.

**Figure 5:**
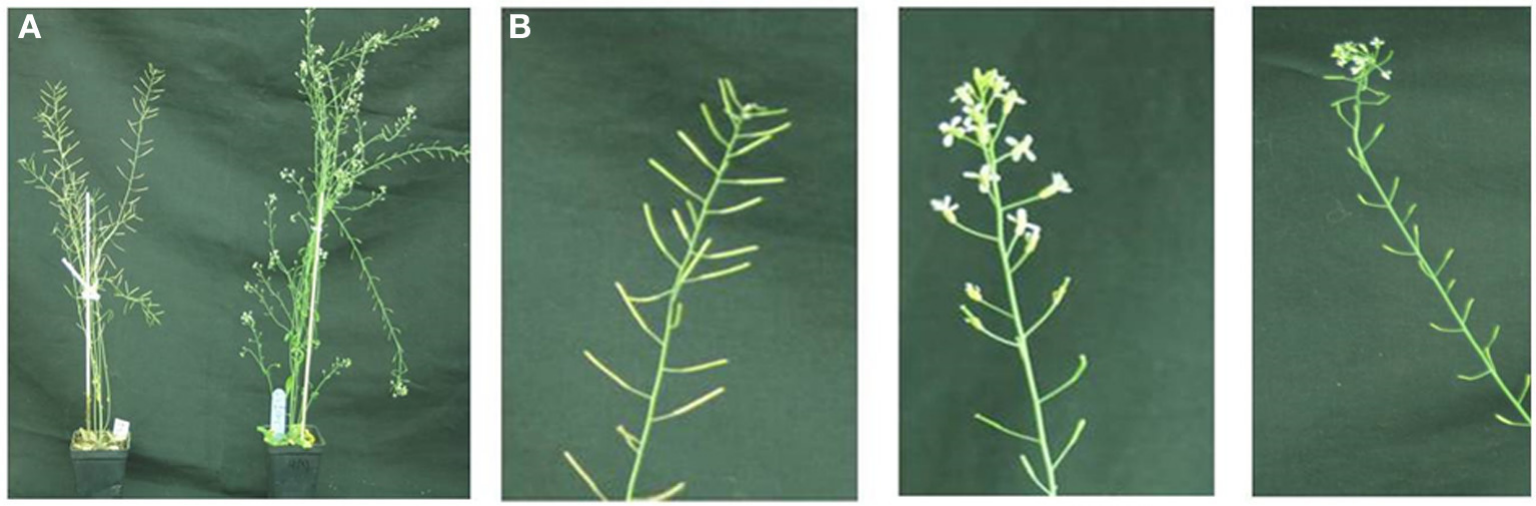
**Ectopic expression of ScFT1-1 affects flowering time and silique development. (A)** Comparison of development timing of ScFT1-1 (right) with Col-0 wild-type plant (left). **(B)** Close-up at the siliques from Col-0 (left), ScFT1-1 flowers (middle), and abnormal siliques (right).

In addition to later flowering, all ScFT1 over-expressing lines often exhibited defects in floral organ formation. Unlike *ScTFL1* lines, where the latest flowering phenotype was associated with defects in reproductive structures, the *ScFT1* over-expressing line with the most severe reproductive abnormalities was ScFT1-1, which consistently had a high number of sterile flowers and formed abnormally shorter siliques (Figure 5A). Furthermore, most siliques had abnormal development, leading to poor seed set and mostly sterile plants. In ScFT1 transgenic lines, open flowers did not self-fertilize and siliques did not develop from fertilized carpels, which may explain the shorter siliques (Figure 5B).

These results suggest that *ScFT1* may be involved in meristem activities that control flowering time and production of fertile organs, although further analysis is required to understand the effects of *ScFT1* overexpression in meristem development. Other studies have reported that loss of *FT-like* function caused meristem-associated abnormalities (Bohlenius et al., 2006; Shalit et al., 2009; Krieger et al., 2010; Danilevskaya et al., 2011; Navarro et al., 2011).

### Yet to be characterized ScFT-like genes may be involved in sugarcane floral induction

**Figure 6:**
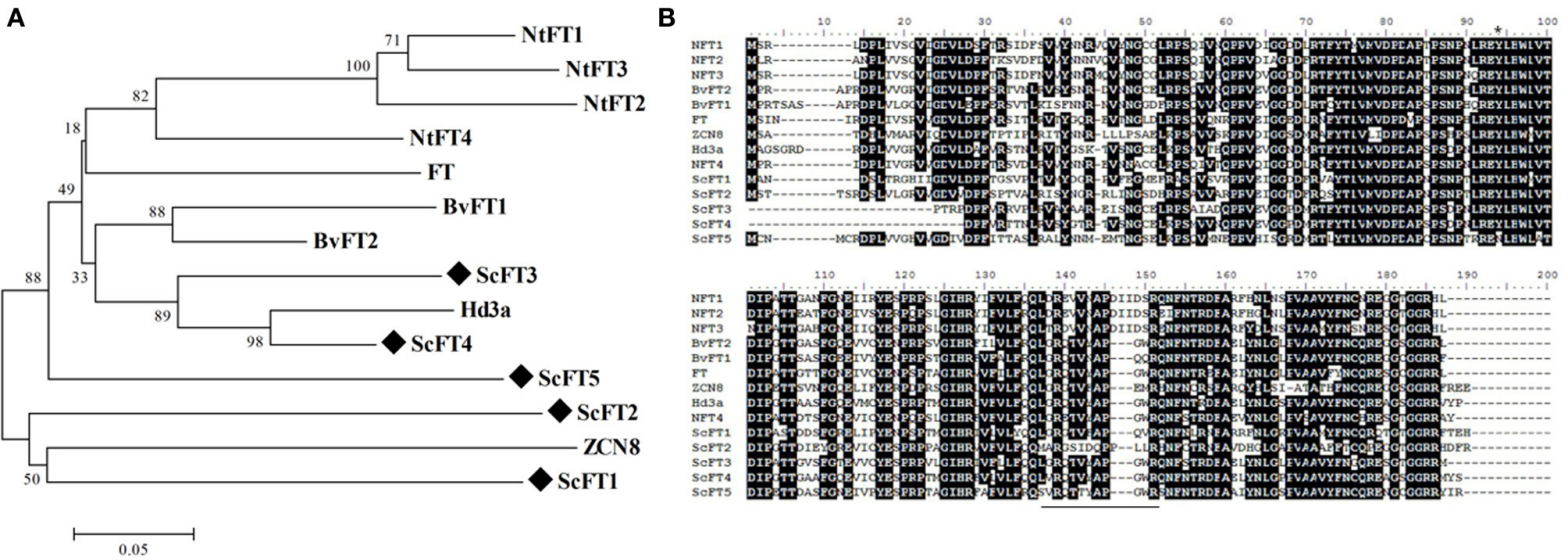
**Sequence analysis of ScFT protein candidates with other FT homologs. (A)** Amino acid sequences from different species were aligned to five ScFT-like candidates using ClustalW. **(B)** Phylogenetic tree of FT homologs. Bootstrap values from 1000 replications were used to assess the robustness of the tree. Sugarcane ScFT-like candidate genes are highlighted by a diamond symbol (♦) and FT homolog from related species are deposited at the Genbank database. A black line highlights segment **(B)**. Accession numbers: BvFT1 (ADM92608.1), BvFT2 (ADM92610.1), NtFT1 (AFS17369.1), NtFT2 (AFS17370.1), NtFT3 (AFS17371.1), NtFT4 (AFS17372.1), FT (BAA77838.1), Hd3a (BAB61030.1), ZCN8 (ABX11010.1).

*ScFT1* is the only full-length *FT-like* candidate we were able to isolate from the sugarcane genome. Four other incomplete sequences were identified in the sugarcane EST database (SUCEST), which we designate *ScFT2*, *ScFT3*, *ScFt4*, and *ScFT5*. Of the candidates that were analyzed, *ScFT2* is most closely related to *FT-like* candidates maize *ZCN8* and *ScFT1*. The *ScFT3* and *ScFT4* putative homologs clade with all floral promoter *FT-like* genes, *Hd3a*, *FT*, and *BvFT1* (Figure 6A), indicating that we cannot rule out the possibility that one or both of them may act as florigen in sugarcane. Functional characterization of these candidates will enlighten this hypothesis. Phylogenetic analysis indicates that *ScFT3* and *ScFT4* clade with floral promoters, given the high degree of similarity of segment B compared to other FT floral promoting proteins (Figure 6B).

## Discussion

### ScTFL1 maintains meristem indeterminacy in arabidopsis, suggesting a similar role in sugarcane

Although extreme late flowering is a negative agronomic trait in many crops, it is of great advantage in commercial sugarcane plants, where a non-flowering phenotype is a highly desirable trait that is the objective of many sugarcane-breeding programs (Berding and Hurney, 2005; Van Heerden et al., 2010). Maintaining sugarcane plants in a vegetative state prevents the loss of sugar accumulation in the stalks that would result from precocious flowering, especially in the tropics where day-length is inductive for floral transition throughout the year.

As a first step in elucidating the molecular mechanisms that control flowering in sugarcane we isolated *FT/TFL1* gene family homologs of key flowering time genes first characterized in Arabidopsis. Two of these genes, *ScTFL1* and *ScFT1*, were analyzed for their role in flowering by ectopic expression in Arabidopsis. We have validated this technique previously by showing that over-expression of another monocot flowering gene, the maize *FT-like* gene *ZCN8*, had a dramatic effect on Arabidopsis flowering (Lazakis et al., 2011). Similarly ectopic expression of *FT/TFL* related genes have been demonstrated for diverse plant species (Jensen et al., 2001; Nakagawa et al., 2002; Mimida et al., 2009; Pin et al., 2010; Karlgren et al., 2011; Klintenas et al., 2012).

Late flowering was observed in Arabidopsis plants over-expressing *ScTFL1*. This suggests that *ScTFL1* acts by extending the duration of growth phases and maintenance of the inflorescence meristem in sugarcane. The latest flowering *ScTFL1* overexpressing plants also had abnormal floral organ structures, which may be due to an imbalance between *TFL1* and *AP1* expression. This leads to floral reversion to vegetative structures, triggering the appearance of floral buds inside the aerial rosettes, resulting in enhanced indeterminate growth. In *wild-type* plants, *AP1* down-regulates *TFL1* in floral meristems and, in turn, *TFL1* maintains indeterminate growth of the vegetative center (Ratcliffe et al., 1999). Although complete inhibition of flowering has not been observed in any single mutants in Arabidopsis, this is not the case for double mutants. Floral transition is never completed in *pennywise* and *pound-foolish* (*pny pnf*) double mutants (Smith et al., 2004). Loss of both of these duplicate BELL homeobox genes results in ectopic *TFL1* expression at high levels in the vasculature, the same site of *FT* expression. This indicates that ectopic overexpression of *TFL1* can result in a non-flowering phenotype.

The altered architecture of the ScTFL1-41 is similar to that observed in the Arabidopsis late-flowering ecotype, Sy-0, which also has aerial rosettes formation in flowering stems (Grbic and Bleecker, 1996). Aerial rosette formation is often related to loss of floral meristem identity genes responsible for signal transduction of the *AP1* gene, an integrator of all flowering pathways that determines floral organ formation. By the time *AP1* is expressed, floral determination is initiated and plants continue to flower independent of environmental signals (Hempel et al., 1997). Grbic and Bleecker (1996) suggested that this phenotype is a result of the interaction of two main dominant genes, *AERIAL ROSETTE (ART)* and *ENHANCER OF AERIAL ROSETTE (EAR)*. Mutant phenotypes of *aerial rosette 1* (*art1)* are a consequence of a delay from the vegetative (V) to reproductive (R) phase transitions, resulting in the formation of a new type of metamer consisting of V1 → V2^*^ → R^*^ → R, in which aerial rosettes are formed by the V2^*^ stage (Poduska et al., 2003). Similar to this, 35S::*TFL1* plants also have a prolonged vegetative phase and produce aerial rosettes (Ratcliffe et al., 1998, 1999). Aerial rosette formation also was reported when another *BELL* gene, *ATH1*, was over-expressed in Arabidopsis (Proveniers et al., 2007).

Increased axillary branching of the *ScTFL1* transgenic plants is similar to the effects observed when other *TFL1* homologs are over-expressed in Arabidopsis. It was suggested that this phenotype may be a consequence of interaction of TFL1 with hormones, since plant hormones such as auxin, cytokinin and strigolactone play a role in the branching and outgrowth of plants (McSteen, 2009; Danilevskaya et al., 2010). The TFL1 protein complex was reported previously, and the external loop seems to be the site for co-repressors/co-activators to bind and trigger developmental responses. Nevertheless, these co-repressors/co-activators have not yet been identified (Taoka et al., 2013), raising the possibility that plant hormone activity could be connected to the mechanisms by which ScTFL1 results in the observed phenotypes.

Mechanisms of *TFL1* function are less clear than that of *FT*; moreover *TFL1* function may vary among annual and perennial plants. *TFL1* was reported to act in an age-dependent flowering pathway in the perennial *Arabis alpina*. In these plants, *AaTFL1* is responsible for the maintenance of vegetative growth of young plants, even under inductive conditions, preventing all axillary meristems from becoming determined. As the shoot ages, *AaTFL1* sets an increasing flowering threshold and the plant is able to develop perennial traits (Wang et al., 2011). In perennial ryegrass, the *TFL1* homolog *LpTFL1* is up-regulated in the apex once the temperature and day-length increases, allowing for lateral branching, and consequently, the promotion of tillering (Jensen et al., 2001). It is possible that *ScTFL1* acts in a similar manner in perennial sugarcane, perhaps explaining the expression of this gene in leaves surrounding the peripheral regions of the meristem of vegetatively growing plants. Further studies of the expression pattern of *ScTFL1* at the shoot apex and surrounding developing leaves of mature flowering plants may provide insights about this possibility.

### *ScFT1* may control flowering time and inflorescence formation in sugarcane

Evolutionary analysis of the PEBP family suggests that *FT*-like and *TFL1*-like subfamilies arose from a common *TFL1*-like ancestor, and that the *FT*-like floral promoter evolved within the angiosperm clade (Karlgren et al., 2011; Klintenas et al., 2012). Therefore it is possible that floral repressor activity of *FT*-like genes persists among angiosperms, as has been reported for tobacco (Harig et al., 2012) and sugar beet (Pin et al., 2010).

The present work suggests that *ScFT1* functions as a floral repressor in sugarcane. Strikingly, expression of *ScFT1* varies under non-inductive and inductive conditions. Under non-inductive long-day conditions, *ScFT1* is expressed in both immature leaves and the apical meristem region, but is not detected in mature leaves. Under inductive short day conditions, however, *ScFT1* is expressed in mature leaves, which are the source of the florigen signal. Together with the late flowering phenotype observed in the overexpressing Arabidopsis lines, this could indicate that *ScFT1* is associated with an anti-florigen signal that originates in mature leaves under floral inductive conditions to counter-balance the florigen signal.

The effect of *ScFT1* overexpression on silique development is similar to that observed in Arabidopsis *dyt1* mutants; *DYSFUNCTIONAL TAPETUM1* (*DYT1*) is involved in tapetum differentiation and function and, without functional *DYT1*, normal anther development is interrupted, generating plants with very small siliques (Zhang et al., 2006). *BEL1* and *SHORT INTEGUMENT* (*SIN1)* control ovule development in Arabidopsis as *bel1* mutants transform ovule integuments into carpels due to ectopic expression of *AGAMOUS* (*AG*) in these tissues (Ray et al., 1994). Alterations of *bel1* mutants include increased axillary buds, delayed senescence and short abnormal siliques formation, similar to what we observe in *ScFT1* plants (Robinson-Beers et al., 1992). Loss of *SIN1* also affects flowering time, resulting in an increased number of rosette leaves and coflorescence branches. *SIN1* is epistatic to *TFL1* and may act in an independent pathway to suppress, at least in part, the *tfl1* phenotype (Ray et al., 1996).

All FT-like proteins involved in floral promotion have a conserved segment B region encoded in the fourth exon that is essential for these homologs to act as florigens in diverse plant species (Ahn et al., 2006; Pin et al., 2010; Harig et al., 2012). Segment B of ScFT1 varies in three amino acid residues compared to the FT and Hd3a floral proteins. In sugar beet variation of three amino acids in segment B of two FT-like proteins is sufficient for them to act antagonistically (Pin et al., 2010). Comparison of partial sequences of several ScFT-like genes from the sugarcane EST database (SUCEST) indicates that ScFT3 and ScFT4 candidates may be involved in the floral promotion, considering the sequence conservation and phylogenetic relationship to FT-like homolog subfamilies. Full-length transcripts need to be characterized to evaluate the effect of sequence plasticity and divergence of functions of sugarcane *FT-like* genes.

Recent discoveries suggest that FT-like proteins act not only as floral repressors but also in diverse developmental events, such as potato tuberization (Navarro et al., 2011), seasonal control of growth cessation in poplar trees (Bohlenius et al., 2006), termination of meristem growth and fruit yield in tomato (Shalit et al., 2009; Krieger et al., 2010), plant architecture in maize (Danilevskaya et al., 2011), and stomatal control in Arabidopsis (Kinoshita et al., 2011). Together these different activities raise a fundamental question about whether FT-like proteins function as versatile mobile signals orchestrating diverse processes in plant development rather than solely acting as a florigen (Taoka et al., 2013).

## Author contributions

Carla P. Coelho and Joseph Colasanti designed the experiments and organized the manuscript. Carla P. Coelho and Mark A. A. Minow performed the experiments. Antonio Chalfun-Júnior and Joseph Colasanti edited the manuscript. All authors discussed the results and implications and commented on the manuscript at all stages.

### Conflict of interest statement

The authors declare that the research was conducted in the absence of any commercial or financial relationships that could be construed as a potential conflict of interest.

## Supplementary material

Supplemental Figure 1 **Expression analysis of the transgenes in Arabidopsis independent lines. (A)** *ScTFL1* expression relative to Arabidopsis *ACTIN8* expression in the lines ScTFL1-5, ScTFL1-6, ScTFL1-11, and ScTFL1-41. **(B)** *ScFT1* expression relative to *ACTIN8* expression in the lines ScFT1-1, ScFT1-2, ScFT3, and ScFT4. Error bars denote relative quantity maximum and minimum values from triplicate biological samples, with each sample a pool of five plantlets.

Supplemental Figure 2 **Ectopic *ScTFL1* expression affects flowering in four independent lines of transgenic Arabidopsis, under long-day conditions after 43 days. (A)** 35S::ScTLF1-5; **(B)** 35S::ScTFL1-6; **(C)** 35S::ScTFL1-11; **(D)** 35S::ScTFL1-41. Transgenic plants are on the right plants and Col-0 wild-type plants are on the left in all panels.
