# Supplementary figures and images for: Putative sugarcane FT/TFL1 genes delay flowering time and alter reproductive architecture in Arabidopsis
Source: Front Plant Sci. 2014 May 26;5:221. doi: 10.3389/fpls.2014.00221 (PMC4033272; doi:10.3389/fpls.2014.00221)

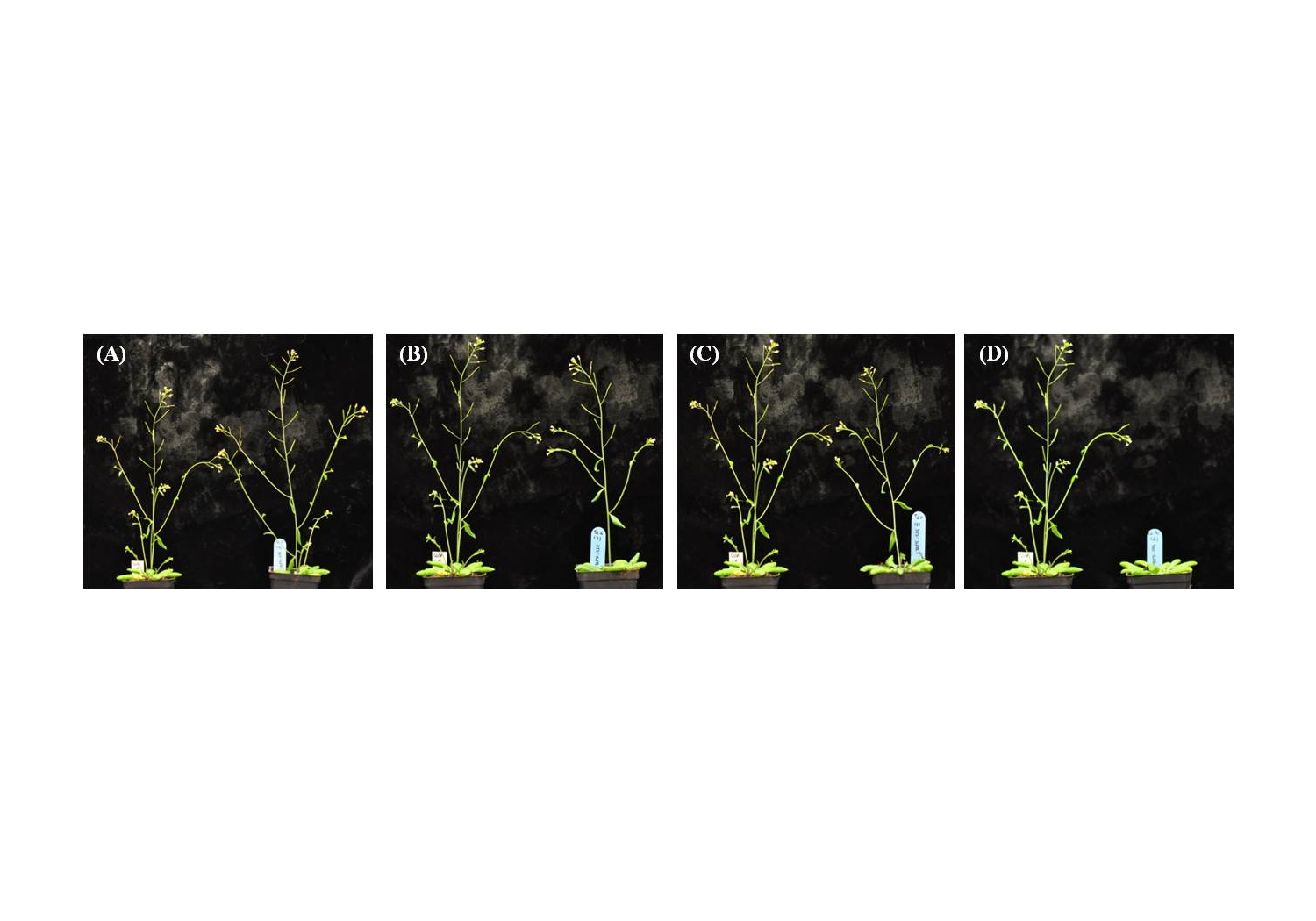

Supplement: Supplemental Figure 1 — Expression analysis of the transgenes in Arabidopsis independent lines. (A) ScTFL1 expression relative to Arabidopsis ACTIN8 expression in the lines ScTFL1-5, ScTFL1-6, ScTFL1-11, and ScTFL1-41. (B) ScFT1 expression relative to ACTIN8 expression in the lines ScFT1-1, ScFT1-2, ScFT3, and ScFT4. Error bars denote relative quantity maximum and minimum values from triplicate biological samples, with each sample a pool of five plantlets. [file Presentation1.ZIP › 82730_Supp Fig 2.JPEG]

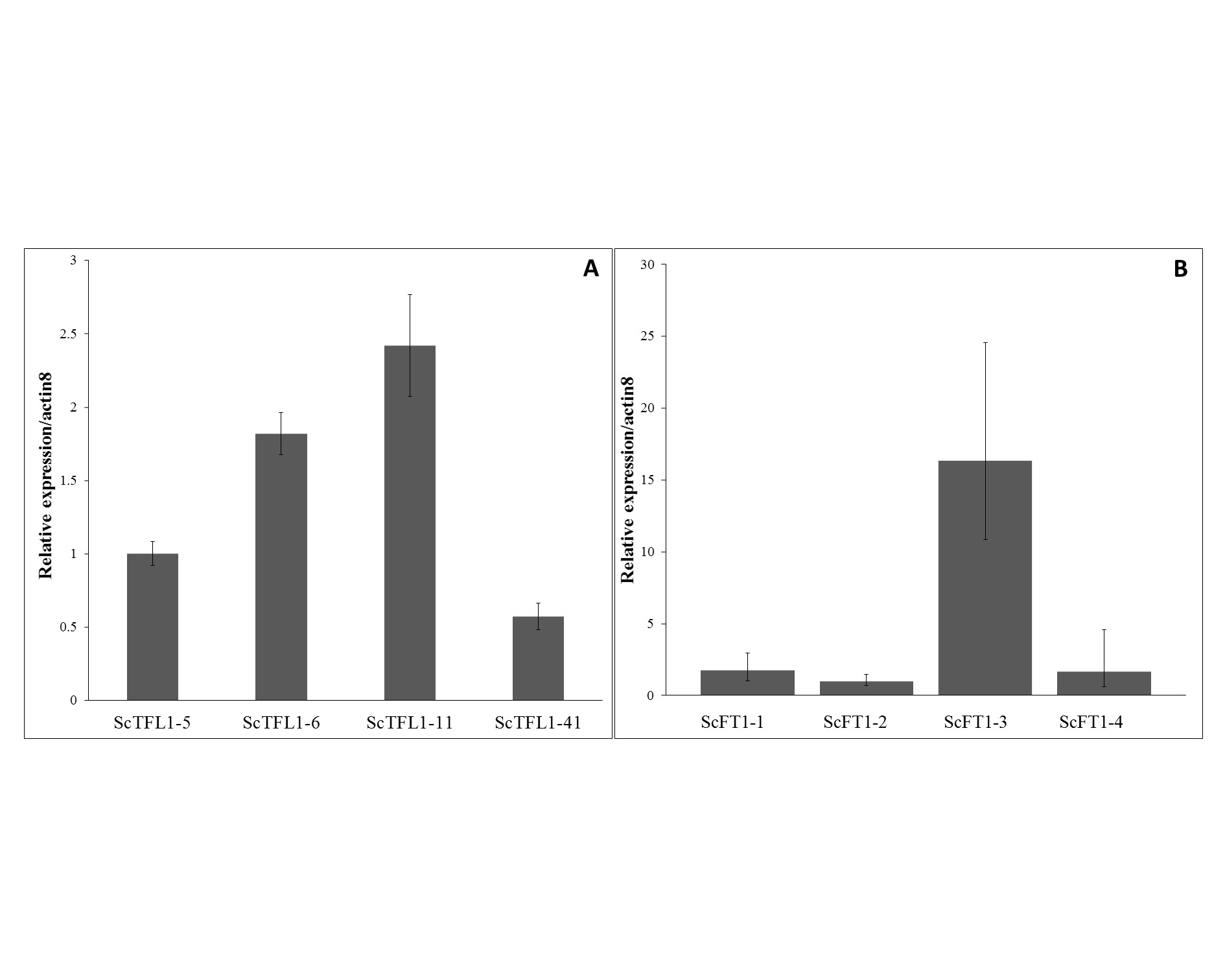

Supplement: Supplemental Figure 1 — Expression analysis of the transgenes in Arabidopsis independent lines. (A) ScTFL1 expression relative to Arabidopsis ACTIN8 expression in the lines ScTFL1-5, ScTFL1-6, ScTFL1-11, and ScTFL1-41. (B) ScFT1 expression relative to ACTIN8 expression in the lines ScFT1-1, ScFT1-2, ScFT3, and ScFT4. Error bars denote relative quantity maximum and minimum values from triplicate biological samples, with each sample a pool of five plantlets. [file Presentation1.ZIP › 82730_Supp Fig 1.JPEG]
